# Investigation of the leaching mechanism of NMC 811 (LiNi_0.8_Mn_0.1_Co_0.1_O_2_) by hydrochloric acid for recycling lithium ion battery cathodes

**DOI:** 10.1039/c9ra06686a

**Published:** 2019-11-26

**Authors:** Wen Xuan, Akira Otsuki, Alexandre Chagnes

**Affiliations:** Université de Lorraine, CNRS, GeoRessources F-54000 Nancy France alexandre.chagnes@univ-lorraine.fr

## Abstract

This paper investigates the reactions involved when LiNi_0.8_Mn_0.1_Co_0.1_O_2_ (NMC 811), which is one of the most promising positive electrodes for the next generation of lithium-ion batteries, is leached by hydrochloric acid. This study shows that the leaching behaviour of lithium is quite different than those observed for nickel, cobalt and manganese contained in NMC 811 since lithium dissolution is faster than those observed for nickel, cobalt and manganese. Analysis of leaching kinetic data evidenced that NMC 811 dissolution occurs in two steps. In the first step, NMC is transformed into a new phase which contains less lithium (2.8 < *n* < 3.6): 1^st^ step: 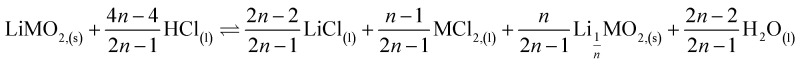 where M = Ni, Mn, Co. In the second step, the new phase is dissolved (limiting step): 2^nd^ step: 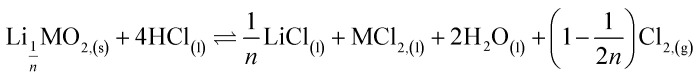. Finally, the overall reaction of NMC 811 leaching by hydrochloric acid can be written as: 2LiMO_2,(s)_ + 8HCl_(l)_ ⇌ 2LiCl_(l)_ + 2MCl_2,(l)_ + 4H_2_O_(l)_ + Cl_2,(g)_.

## Abstract 2

This paper investigates the reactions involved when LiNi_0.8_Mn_0.1_Co_0.1_O_2_ (NMC 811), which is one of the most promising positive electrodes for the next generation of lithium-ion batteries, is leached by hydrochloric acid.

## Graphical abstract

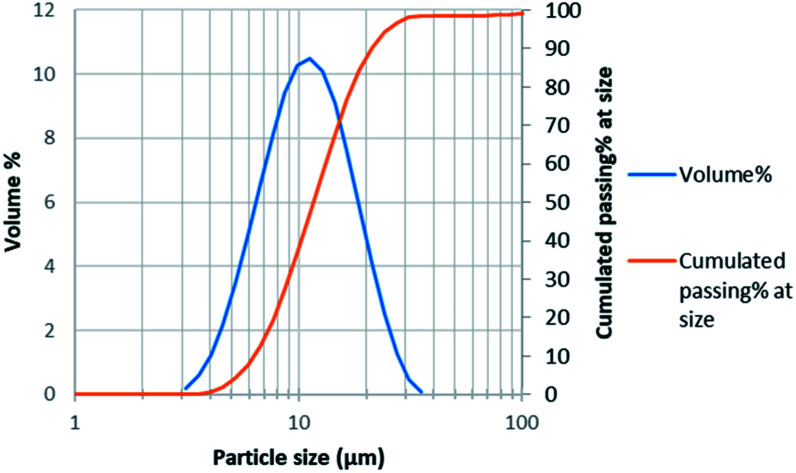

## 1 Introduction

Since 1991, lithium-ion batteries (LIBs) have been widely used in electronic devices and electric vehicles, because it can provide high energy and power per unit of battery weight.^1,2^ Concerns have been raised as the increasing demand of raw materials may not be easily satisfied, especially for countries where resource availability is limited. On the other hand, inappropriate disposal of large amounts of spent LIBs can lead to serious environmental and safety issues.^3,4^ In addition, lithium and cobalt recovery from primary and spent LIBs is an important topic as Co and Li are classified as critical energy materials by the US, EU and UN. Therefore, recycling of spent LIBs presents significant environmental and economic importance.^5^ However, in many places, the word “recycling” generally only refers to collection, sorting and dismantling of battery wastes, and does not include the further process of raw material recovery from these wastes.^6^

Commercial LIBs usually use various types of Li containing oxides and phosphate as cathode materials, such as LiCoO_2_, LiMn_2_O_4_, LiFePO_4_, LiNi_1−*x*−*y*_Mn_*x*_Co_*y*_O_2_ (NMC, or sometimes also called NCM), *etc.*

Ternary composites Li(Ni_1−*x*−*y*_Mn_*x*_Co_*y*_)O_2_ are widely used as cathode material in LIBs. In these electrodes, nickel changes its oxidation states during charge–discharge cycling of the battery due to lithium insertion–deinsertion (Ni^2+^ ↔ Ni^4+^). High content of nickel results in high capacity and rate capability but also in poor safety. Manganese dominates the safety and cycle life. Cobalt stabilizes layer structure of materials and also improves rate capability.^7^ During the last years, more and more attention has been paid to LiNi_0.8_Mn_0.1_Co_0.1_O_2_ (NMC 811) since this material is considered a good alternative to LiNi_1/3_Mn_1/3_Co_1/3_O_2_ (NMC 111) for the next generations of LIBs because of the good compromise between its performance and its stability. Furthermore, the use of NMC 811 instead of NMC 111 will undoubtedly reduce cobalt demand and supply risk for this element, which is a critical metal. Nevertheless, only few studies are devoted to investigate NMC 811 recycling while more papers concerns the development of recycling processes for the recovery of metals from NMC 111.^8–10^

Hydrometallurgy appears as the good technology to extract valuable metals from spent LIBs because it can recover all metals contained in spent LIBs including lithium unlike pyrometallurgy.^11^ This process usually involves acid leaching to dissolve the cathode material, followed by solvent extraction or precipitation to separate each metal. Hydrochloric acid, nitric acid, sulfuric acid and several organic acids like lactic acid, citric acid, formic acid, and tartaric acid were tested in previous works^12,13^ while many complete flowsheets implementing chemical operations such as leaching and solvent extraction were proposed.^14^

Sulfuric acid has been extensively studied in the literature for dissolving cathode materials. Leaching efficiency has been improved by adding a reducing agent like H_2_O_2_ to reduce Co(iii) into Co(ii) according to the following reaction:^15^2LiCoO_2_ + 3H_2_SO_4_ + H_2_O_2_ = Li_2_SO_4_ + 2CoSO_4_ + 4H_2_O + O_2_

LiCoO_2_ can also be fully dissolved by sulfuric acid without the use of any reducing agent according to the following equation:^16^4LiCoO_2_ + 6H_2_SO_4_ = 2Li_2_SO_4_ + 4CoSO_4_ + 6H_2_O + O_2_

In this case, leaching has to be performed under harder conditions (3 mol L^−1^ H_2_SO_4_ at 70 °C) than with H_2_SO_4_ in the presence of H_2_O_2_. However, H_2_O_2_ is not safe due peroxide formation leading to explosion risks. Ascorbic acid is another class of acid exhibiting reducing properties, which is safe, but industrials prefer using classical reactive because of availability risk. Therefore, hydrochloric acid remains a good alternative towards sulfuric acid and hydrogen peroxide in spite of its corrosive properties and chlorine generation. However, corrosion issues can be solved by using materials with high corrosion resistance such as graphite, titanium and Hastelloy C. The later are very expensive but other cheaper materials can be used like rubber lined steel. Regarding chlorine gas generation during leaching, chlorine can be advantageously used to produce hydrochloric acid that can be recycled in the leaching operation in order to reduce OPEX of the process. Furthermore, the use of hydrochloric acid may pave the way to efficient separation of cobalt, nickel and manganese by solvent extraction while such a separation remains difficult in acidic sulfate media.

In the case of LiCoO_2_, which was the most studied cathode material, it is usually stated that the leaching mechanism by HCl can be described by the following equation:^17^

1 2LiCoO_2,(s)_ + 8HCl_(l)_ ⇌ 2LiCl_(l)_ + 2CoCl_2,(l)_ + 4H_2_O_(l)_ + Cl_2,(g)_

In the case of NMC 111, some authors suggested that cathode dissolution in the presence of reductant involves two dissolution steps with different mechanisms and limitations depending on the nature of the acid and the cathode, and that transition metals leaching efficiency has a threshold value around 40%.^18^ Further studies on sulfuric acid leaching of NMC 111 suggested that the leaching process leads to the formation of a well-defined core–shell structure inducing an enrichment in manganese onto the particle surface.^19^

Most of the studies reported in the literature about leaching of cathode materials from LIBs concern NMC 111 and most of these studies focus on the use of sulfuric acid as leaching agent whereas no paper has recently studied NMC 811 leaching in acidic chloride media in spite of its high potentialities for the next generations of LiBs.

For the first time, this paper investigates NMC 811 leaching by hydrochloric acid and identifies clearly the corresponding reactions.

## 2 Experimental

### 2.1 Materials and methods

Hydrochloric acid (37% weight) was supplied by Sigma Aldrich. NMC 811 was supplied by Xiamen Tob New Technology Co., LTD. Its chemical composition was determined by ICP-AES (Table 1).

**Table 1:** Chemical composition of NMC 811

	Element				
	Li	Ni	Mn	Co	O
Weight%	7.36	46.01	5.40	6.28	34.95
Molar ratio	1.00	0.74	0.09	0.10	2.06

Elemental analysis confirmed that the composition of the cathode material provided by Xiamen Tob New Technology Co corresponds to NMC 811 even if it appears that nickel content is slightly lower than expected: LiNi_0.74_Mn_0.09_Co_0.10_O_2.06_ instead of LiNi_0.8_Mn_0.1_Co_0.1_O_2_.

**Fig. 1:**
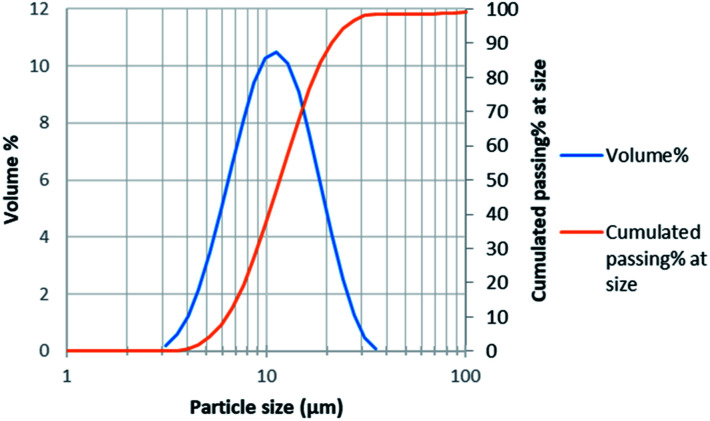
Particle size distribution of NMC 811.

Particle size distribution measurement was performed by laser diffraction using Malvern Mastersizer 3000. Average particle size was 11 μm with *d*_50_ = 12 μm and *d*_80_ = 17 μm (Fig. 1).

The leaching experiments were performed in a homemade double-jacketed 1L-glassware reactor thermostated with water heated by means of a Lauda thermostat. Diluted acid was added into the reactor. It was agitated by a mechanical steel agitator with PTFE coating at 230 rpm, and regulated to the desired temperature before introducing NMC 811 powder. Samples of about 10 mL were withdrawn regularly from the reactor and immediately filtered by a syringe filter with pore size of 200 μm before further elemental analyses by Microwave Plasma-Atomic Emission Spectrometry (Agilent 4210 MP-AES). The measured cation concentrations were afterwards used to calculate the coefficients in the leaching reaction. Chloride concentrations were measured by ionic chromatography by means of ICS-900 Dionex equipped with IC Dionex™ IonPac™ AS22 type separation column.

The filter cake was dried before further characterisation by X-Ray Diffraction (XRD) after mixing the sample with 10 wt% finely ground pure calcite. XRD spectra were recorded between 2*θ* = 3° and 2*θ* = 90° by means of the diffractometer D8 Advance Bruker (cobalt anode, *λ*_Co_Kα__ = 1.79 Å) equipped with a linear detector LynxEye.

## 3 Results and discussion

### 3.1 Proposal of a leaching mechanism

Wang *et al.*^20^ showed that the use of 4 mol L^−1^ hydrochloric acid at 80 °C and a solid/liquid ratio S/L = 2 g L^−1^ recovered most of Co, Mn, Ni and Li from LiCoO_2_, LiMn_2_O_4_ and LiNi_1/3_Mn_1/3_Co_1/3_O_2_. The same experimental conditions were carried out to leach NMC 811.

Surprisingly, NMC 811 sample was totally dissolved in less than 10 minutes and the reaction was too fast to take any sample. Thus, all of the following experiments were performed at 25 °C to obtain reliable and accurate data. A series of preliminary leaching reactions were performed by varying S/L ratio corresponding to excess acid (S/L = 2 g L^−1^ and 20 g L^−1^ given that the stoichiometric ratio is equal to S/L = 97.3 g L^−1^).

**Fig. 2:**
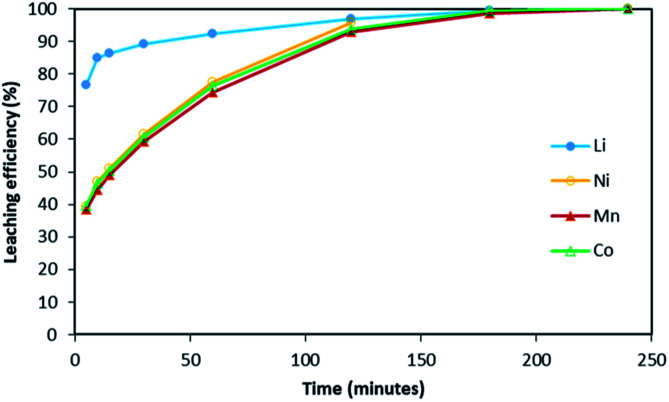
Leaching kinetics of NMC 811 in 4 mol L^−1^ HCl at S/L = 2 g L^−1^ and 25 °C.

**Fig. 3:**
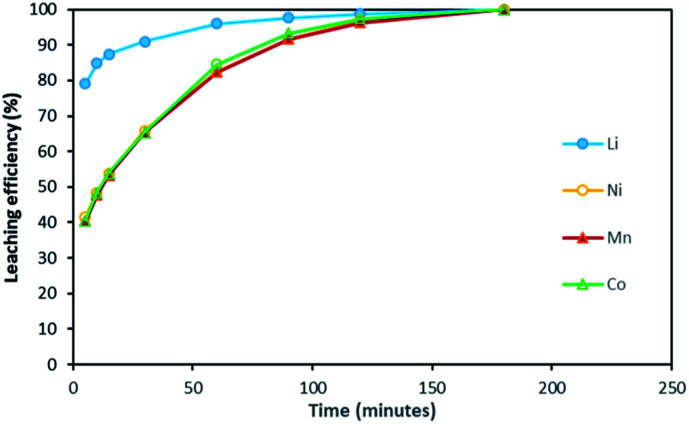
Leaching kinetics of NMC 811 in 4 mol L^−1^ HCl at S/L = 20 g L^−1^ and 25 °C.

Fig. 2 shows it took about three hours to dissolve NMC 811 completely at S/L = 2 g L^−1^. The leaching kinetic of Ni, Mn and Co are almost the same while the leaching rate of Li is twice faster than the other elements at the very beginning. Very similar behaviour was observed at S/L = 20 g L^−1^ as shown in Fig. 3.

**Fig. 4:**
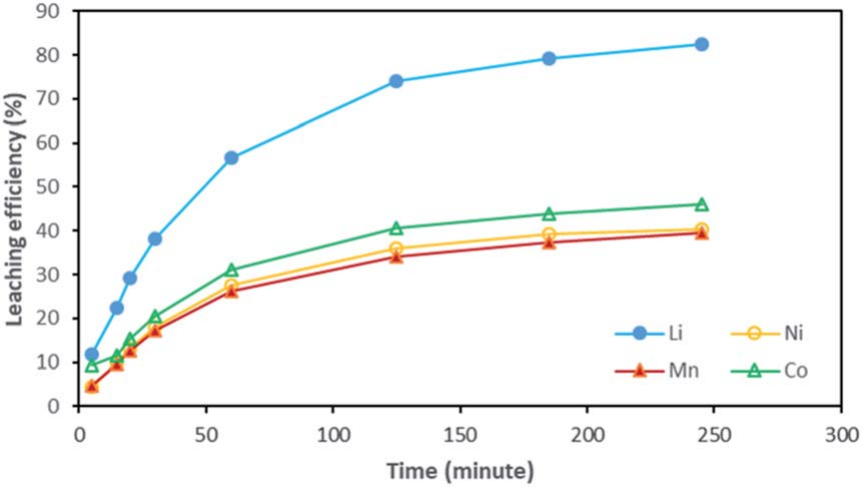
Leaching kinetics of NMC 811 in 1 mol L^−1^ HCl at S/L = 20 g L^−1^ and 25 °C.

Based on the above observations, it seems that the leaching reaction takes place in two steps. However, it is very difficult to take samples during the first step as the kinetic is too fast. Therefore, the same experiment was performed at 25 °C by using 1 mol L^−1^ HCl instead of 4 mol L^−1^ HCl in order to reduce leaching rate (Fig. 4). Once again, Ni, Mn and Co seems to follow the same leaching mechanism since these metals exhibit similar leaching rate throughout NMC 811 dissolution. Li was leached about twice faster than Ni, Mn and Co, until approximately 80% Li and 40% Ni, Mn and Co were leached, which are very close to the starting points of the experiment using 4 mol L^−1^ HCl (Fig. 3). The leaching kinetic in 1 mol L^−1^ HCl (Fig. 4) seems to reveal the initial stage of the leaching kinetics in 4 mol L^−1^ HCl (Fig. 3).

**Fig. 5:**
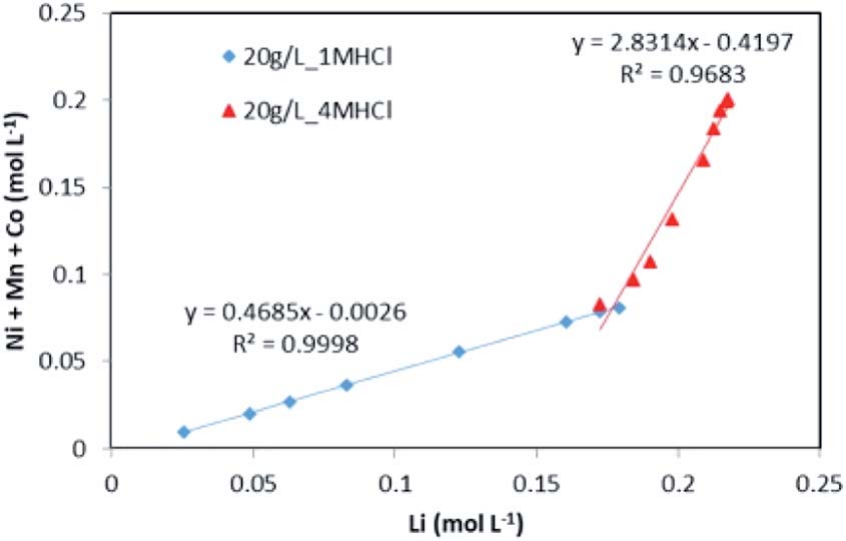
Stoichiometry between the sum of Ni, Mn, Co concentration and Li concentration in leachate at S/L = 20 g L^−1^ during NMC 811 dissolution by 1 mol L^−1^ (blue diamonds) and 4 mol L^−1^ HCl (red triangles) at 25 °C.

The evolution of dissolved Ni, Mn and Co concentrations as a function of dissolved Li concentration in the two previous experiments shows clearly the presence of two distinct stoichiometry ratios around 0.5 and 2.8 (Fig. 5). A transition takes place at the intersection of the two straight lines.

This result further suggests that the leaching reaction takes place in two steps: in the first step, Li is leached twice faster than Ni, Mn and Co whereas Ni, Mn and Co are leached faster than Li in the second step. The first step ends up with the formation of a transition phase containing much less Li, which is dissolved during the second step. The transition phase can be written as:
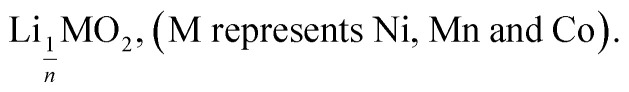


The leaching mechanism may be described by the following two reactions:

First step: 2
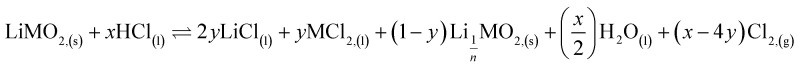


According to the balance on lithium:3
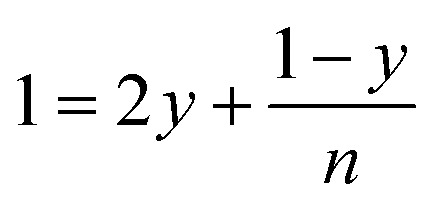


According to the balance on oxygen:4
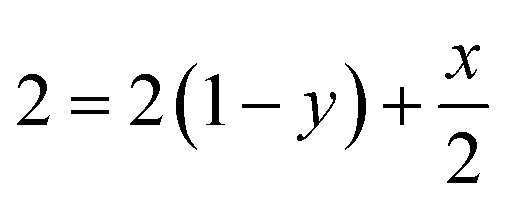


Therefore,5
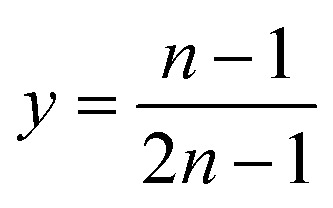
6
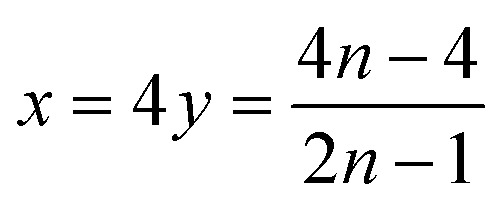


As a result, the first step reaction can be described by the following equation, which does not involve chlorine evolution:7
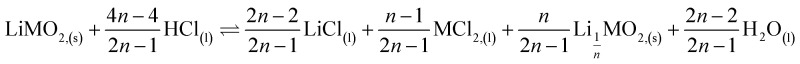


Second step:8
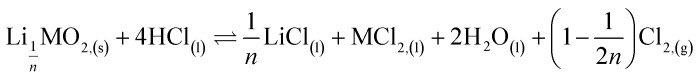


Finally, the overall leaching reaction of NMC 811 by hydrochloric acid can be written as:

9 2LiMO_2,(s)_ + 8HCl_(l)_ ⇌ 2LiCl_(l)_ + 2MCl_2,(l)_ + 4H_2_O_(l)_ + Cl_2,(g)_

Therefore, when there are *a* mol NMC 811 and *ax* mol HCl in the leaching system (NMC/HCl molar ratio = 1/*x*), the maximum leaching efficiency of lithium (LE_Li_) and the other metals contained in NMC 811 (LE_M_ with M = Co, Ni or Mn) can be estimated as follows:

(1) If *x* ≥ 4 then LE_Li_ = LE_M_ = 100%.

(2) If 
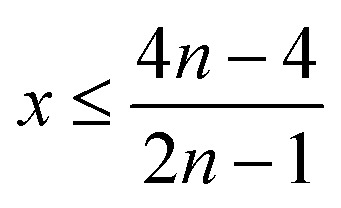
 then10
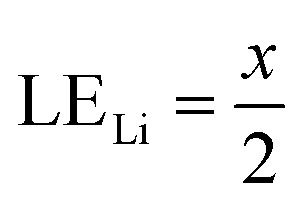
and11
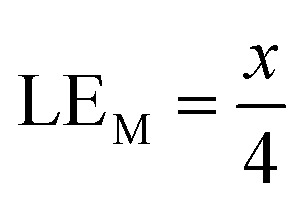


(3) If 
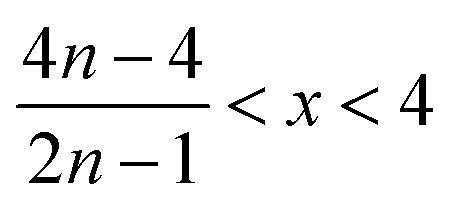
 then12
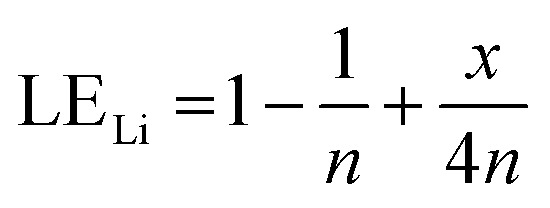
and13
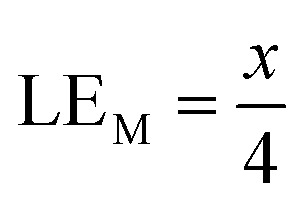


Eqn (9) is in agreement with the leaching reaction of LiCoO_2_ reported elsewhere in the literature [eqn (1)].^13^ On the other hand, this is the first time that such a two-step mechanism is clearly identified for NMC leaching.

### 3.2 Validation of the mechanism

Fig. 2 and 3 have shown that leaching efficiencies of Li, Ni, Mn and Co can reach 100% when *x* > 4. Further validation of the above mechanism was carried out by means of three approaches. Firstly, a leaching test in excess of NMC 811 was performed to check the stoichiometry. Afterwards, a leaching test in slight excess of HCl was performed to assay chloride consumption, and to investigate phase transformation of the cathode material during leaching by XRD.

#### 3.2.1 In the presence of an excess of NMC 811

**Fig. 6:**
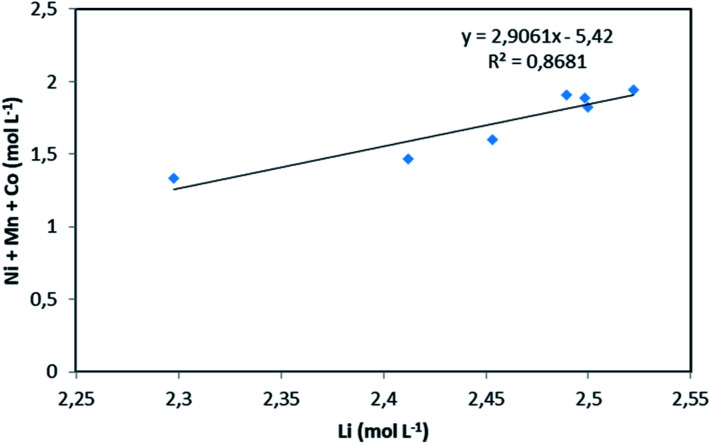
Stoichiometry between the sum of Ni, Mn, Co concentration and Li concentration in leachate at S/L = 120 g L^−1^ during NMC 811 dissolution in 4 mol L^−1^ HCl at 25 °C.

NMC 811 leaching tests were performed in 4 mol L^−1^ HCl at S/L = 120 g L^−1^ and 25 °C, which corresponds to NMC/HCl molar ratio of approximately 1 : 3.23 (*x* = 3.23). The slope of the total concentration of Ni, Mn and Co as a function of lithium concentration in leachate solution throughout the leaching experiment is equal to *n* = 2.91 (Fig. 6).

Therefore, by replacing *n* = 2.91 in eqn (7), the first step of the leaching mechanism can be written as follows:

14 LiMO_2,(s)_ + 1.58HCl_(l)_ ⇌ 0.79LiCl_(l)_ + 0.40MCl_2,(l)_ + 0.60Li_0.34_MO_2,(s)_ + 0.79H_2_O_(l)_

and the second step of the mechanism can be expressed as:

15 Li_0.34_MO_2,(s)_ + 4HCl_(l)_ ⇌ 0.34LiCl_(l)_ + MCl_2,(l)_ + 2H_2_O_(l)_ + 0.83Cl_2,(g)_

**Fig. 7:**
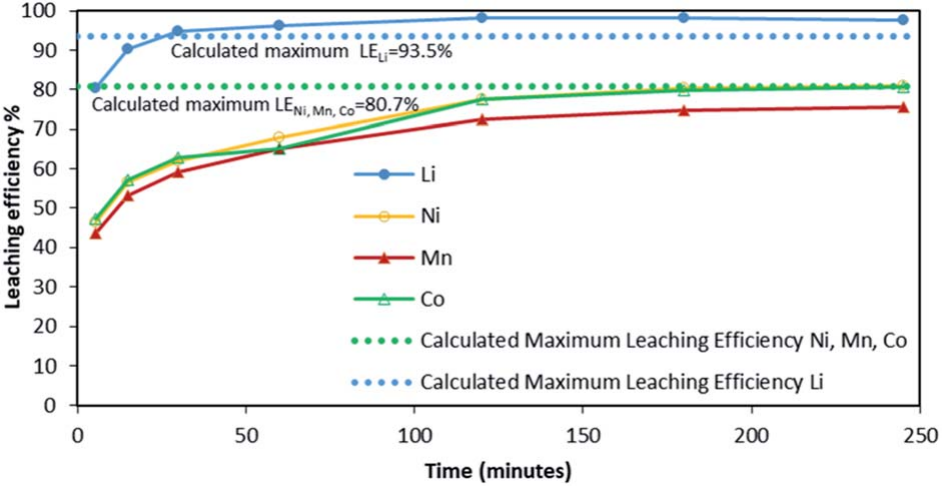
Leaching kinetics of NMC 811 in 4 mol L^−1^ HCl at S/L = 120 g L^−1^ and 25 °C. Dotted line shows the value of the calculated maximum leaching efficiency for Li (blue dotted line) and for Co, Ni and Mn (green dotted line).

Although there is not enough acid for the overall reaction, the acid amount is enough to finalize the first step and form Li_0.34_MO_2_. By applying eqn (12), the maximum leaching efficiency should be LE_Li_ = 93.5% whereas LE_M_ = 80.7% for M = Ni, Mn or Co according to eqn (13). These calculated values are in good agreement with those deduced from leaching experiments as shown in Fig. 7 (experimental maximum leaching efficiencies: LE_Li_ = 98.0%, LE_Ni_ = 81.2%, LE_Mn_ = 75.5%, and LE_Co_ = 81.0).

It is interesting to highlight that high values of S/L ratio do not permit a full dissolution of nickel, manganese and cobalt due to lack of acid. Conversely, lithium was almost completely dissolved as significant lithium dissolution occurs very early in the first step of the leaching process (eqn (7)).

#### 3.2.2 In the presence of an excess of HCl

NMC 811 leaching tests were performed in 4 mol L^−1^ HCl at S/L = 80 g L^−1^ and 25 °C, which is equivalent to NMC/HCl molar ratio of 0.82.

**Fig. 8:**
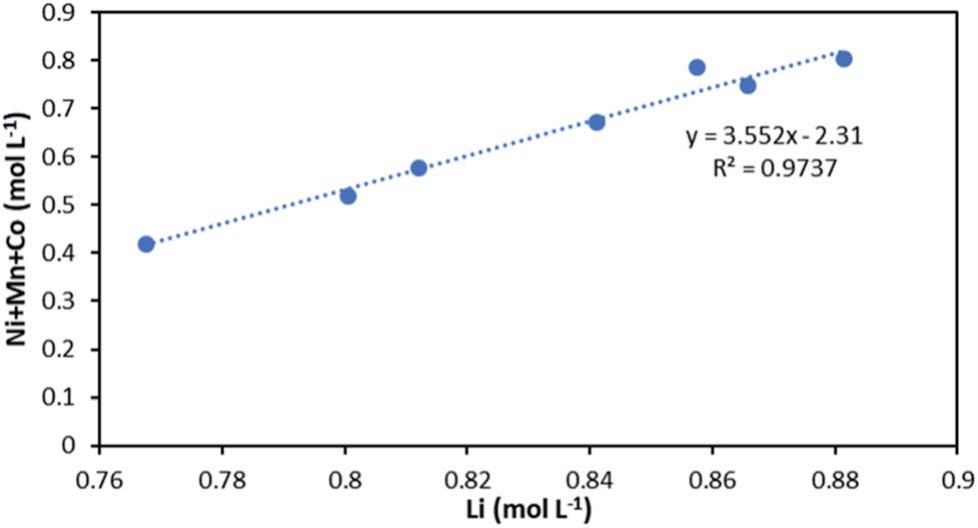
Stoichiometry between the sum of Ni, Mn, Co concentration and Li concentration in leachate at S/L = 80 g L^−1^ during NMC 811 dissolution in 4 mol L^−1^ HCl at 25 °C.

The slope of the total concentration of Ni, Mn and Co as a function of lithium concentration in solution throughout the leaching experiment is equal to *n* = 3.55 (Fig. 8).

Therefore, the following reactions can be deduced from eqn (7):

16 LiMO_2,(s)_ + 1.67HCl_(l)_ ⇌ 0.84LiCl_(l)_ + 0.42MCl_2,(l)_ + 0.58Li_0.28_MO_2,(s)_ + 0.84H_2_O_(l)_

17 Li_0.28_MO_2,(s)_ + 4HCl_(l)_ ⇌ 0.28LiCl_(l)_ + MCl_2,(l)_ + 2H_2_O_(l)_ + 0.86Cl_2,(g)_

**Fig. 9:**
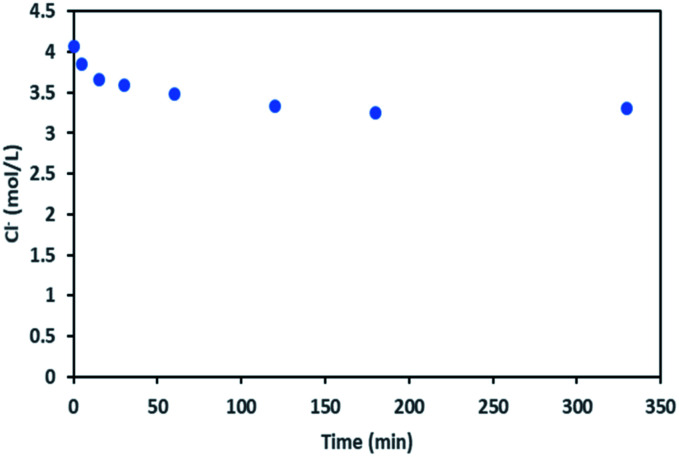
Chloride concentration in leachate at S/L = 80 g L^−1^ during NMC 811 dissolution in 4 mol L^−1^ HCl at 25 °C.

During the leaching process, the chloride concentration decreased continuously (see Fig. 9) as it was transformed to chlorine gas as shown in eqn (17). Chlorine formation was confirmed by the smell of chlorine gas.

**Fig. 10:**
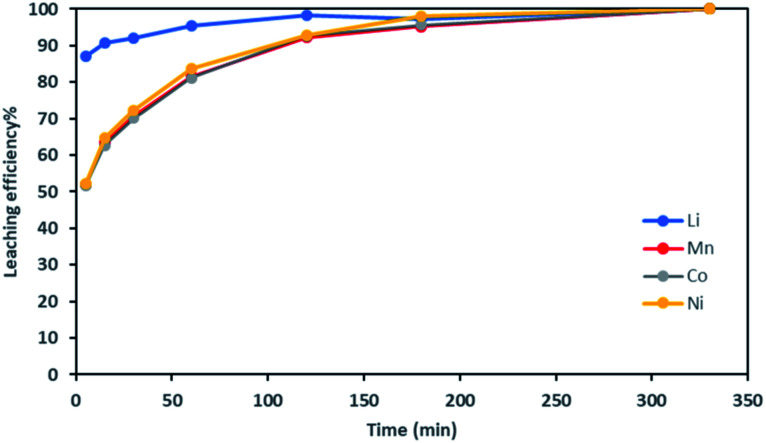
Leaching kinetics of NMC 811 in 4 mol L^−1^ HCl at S/L = 80 g L^−1^ and 25 °C.

**Fig. 11:**
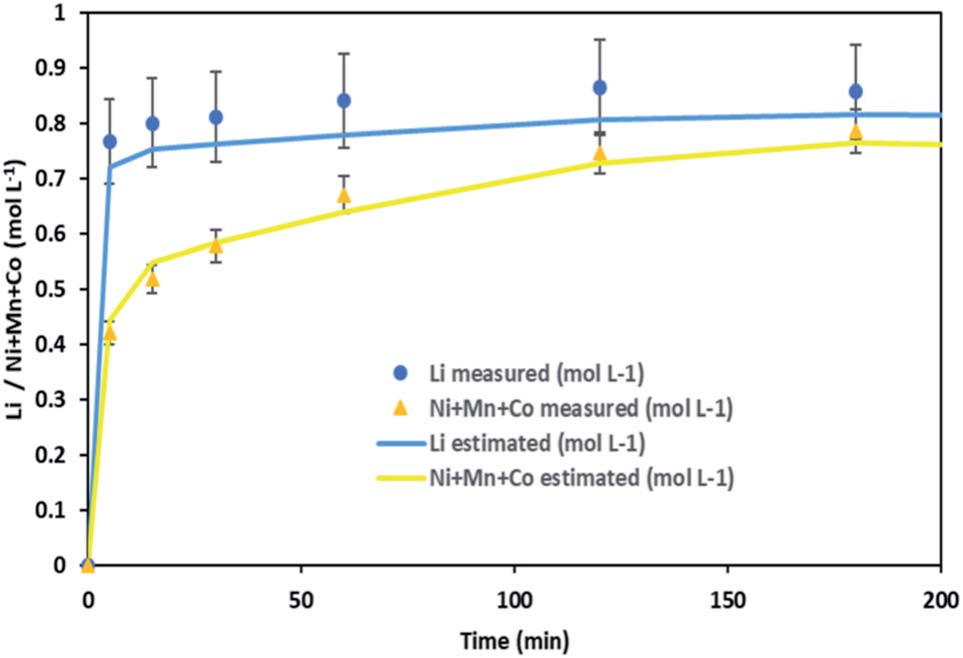
Stoichiometry between the sum of Ni, Mn, Co concentration and Li concentration in leachate at S/L = 80 g L^−1^ during NMC 811 dissolution in 4 mol L^−1^ HCl at 25 °C.

It is thus possible to estimate Li, Ni, Mn and Co concentrations in the leachate by combining chloride consumption and eqn (16) and (17). Fig. 10 shows leaching kinetics of NMC 811 in 4 mol L^−1^ HCl at S/L = 80 g L^−1^ and 25 °C. Fig. 11 compares experimental and calculated concentration of Li, Ni, Mn and Co.

Fig. 11 shows a good agreement between experimental data and calculated ones and justifies the two-step leaching reaction proposed in eqn (7) and (8). It can be observed in Fig. 11 that lithium dissolution is faster than cobalt, nickel and manganese dissolution. This difference in behaviour may be explained by the fact that the mechanism involved in lithium dissolution does not involve any reduction (eqn (14)) unlike cobalt, nickel and manganese (eqn (15)).^21^

**Fig. 12:**
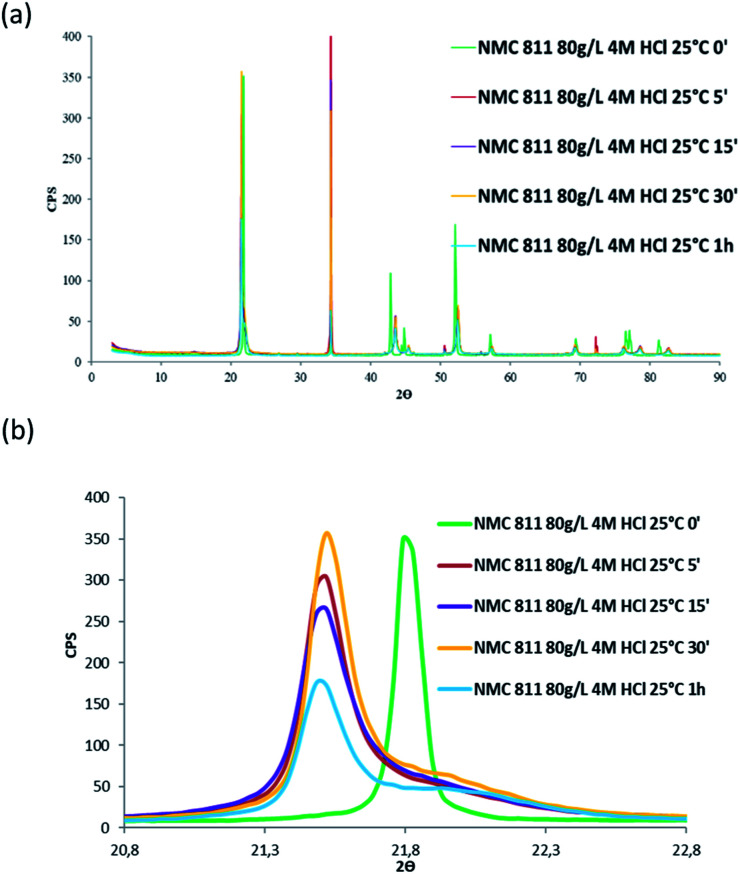
XRD patterns of (a) NMC 811 and (b) zoom on the peak located between 2*θ* = 20.8° and 2*θ* = 22.8° during acid leaching (S/L = 80 g L^−1^; HCl concentration = 4 mol L^−1^, temperature = 25 °C).

Besides, in order to confirm whether a new phase was formed during NMC 811 leaching, intermediate samples were taken during the leaching test. The suspension was firstly diluted 4 times with deionised water in order to slow down the reaction, and then filtered. The filter cake was dried and analysed by XRD (Fig. 12).

**Fig. 13:**
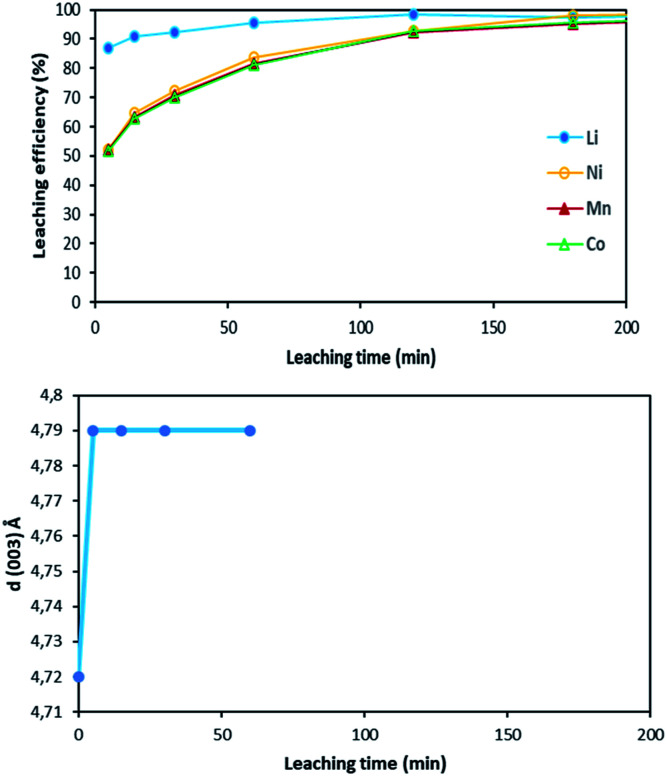
Comparison between leaching kinetics and *d*(003) evolution during leaching (S/L = 80 g L^−1^; HCl concentration = 4 mol L^−1^, temperature = 25 °C).

Fig. 12 shows the main peak located between 2*θ* = 20.8° and 2*θ* = 22.8° shifted towards low 2*θ* values after 5 minutes of leaching due to dilatation of the interplanar space (003). No more variation of the peak position was observed after 5 minutes. Therefore, XRD spectroscopy evidences the formation of a new phase at the beginning of the leaching process. The interplanar spacing of the plan (003) can be calculated from the peak position. The variation of the interplanar space as a function of time confirms the very fast phase transformation within the 5 first minutes of leaching, and it remains stable afterwards (Fig. 13).

Similar shift of (003) peak has been reported in acid delithiation of LiNiO_2_.^22^ Such an increase of the interplanar space of the plan (003) might be due to electrostatic repulsion of Co–O layers caused by lithium deinsertion from NMC 811 as suggested by eqn (16), *i.e.* the first step of the leaching mechanism. At the same time, the Li ions could also be replaced by protons *via* ion-exchange, resulting in M(O,OH)_6_ octahedra in the MO_2_ layers, like the formation of Ni(O,OH)_6_ in the case of acid leaching of LiNiO_2_.^23^

## 4 Conclusions

Hydrochloric acid proves to be efficient in leaching of NMC 811. A new mechanism of acid leaching of NMC 811 was proposed in this study. The mechanism suggests that the leaching reaction takes place in two steps, the first step is a quick phase transformation which forms a new transition phase with less Li, and can be described as:7
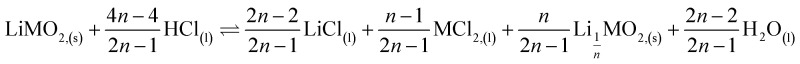
where M represents Ni, Mn or Co, and 2.8 < *n* < 3.6 according to this study. The second step is the dissolution of the transition phase, which can be described by the following equation:8
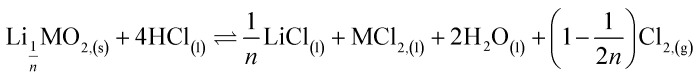


The second step is much slower than the first step. The above two steps lead to the overall reaction equation:

9 2LiMO_2,(s)_ + 8HCl_(l)_ ⇌ 2LiCl_(l)_ + 2MCl_2,(l)_ + 4H_2_O_(l)_ + Cl_2,(g)_

The mechanism meets all experimental data, including both experiments in excess of NMC 811, and experiments in excess of HCl.

Besides, since it does not make difference between nickel, manganese and cobalt, it can very likely be applied to other types of lithium nickel manganese cobalt oxide.

## Conflicts of interest

There are no conflicts to declare.

## Supplementary Material
